# From Multidimensional Management to Mechanistic Insight: A Review of Interventions for Hyperuricemia

**DOI:** 10.3390/ijms27031426

**Published:** 2026-01-30

**Authors:** Quan Sun, Lijun Yin

**Affiliations:** School of Sports, Shenzhen University, Shenzhen 518060, China; 2500201007@mails.szu.edu.cn

**Keywords:** hyperuricemia, multi-dimensional intervention, mechanism

## Abstract

As a major metabolic abnormality following hyperglycemia, hypertension, and hyperlipidemia, hyperuricemia has emerged as a significant global public health issue. The pathological mechanisms of hyperuricemia are complex; it not only directly triggers gout but is also closely associated with various chronic diseases, such as cardiovascular disease, diabetes, and chronic kidney disease, posing a systemic threat to individual health. This article systematically reviews the epidemiological characteristics, pathophysiological mechanisms, clinical consequences, and related risk factors of hyperuricemia, and especially focuses on the research advances and mechanisms of comprehensive intervention strategies, including diet, exercise, pharmacotherapy, and lifestyle modifications. Dietary interventions primarily function by regulating the activity of enzymes and transporters related to uric acid metabolism, ameliorating gut microbiota dysbiosis, and alleviating inflammatory responses. Exercise interventions synergistically improve uric acid homeostasis through multiple mechanisms, including the regulation of purine metabolic enzyme activity and the improvements of body composition, insulin resistance, and oxidative stress. Pharmacotherapy, serving as a core measure for patients with moderate-to-severe conditions, directly lowers serum uric acid levels by inhibiting uric acid production or promoting excretion. Although various intervention modalities exhibit distinct effects in regulating uric acid production, promoting excretion, and improving the metabolic-inflammatory environment, challenges such as significant heterogeneity in individual response and uncertainty regarding long-term efficacy remain prevalent. Furthermore, given the increasing trend toward a younger onset of hyperuricemia, prevention and control strategies targeting children and adolescents require urgent reinforcement. Future efforts should focus on conducting multi-center, large-sample clinical studies with clear mechanisms and establishing individualized health management plans based on population characteristics, thereby promoting the precise prevention and treatment of hyperuricemia.

## 1. Introduction

Hyperuricemia (HUA), as the core manifestation of purine metabolism disorders, has evolved from a condition traditionally associated with gout into a major global public health challenge. Its prevalence continues to rise globally alongside economic development and lifestyle changes, exhibiting significant gender, age, and regional disparities. The global prevalence of HUA is approximately 13.85%, with rates of 21.06% in males and 10.15% in females: the prevalence in adults is 15.17% and in children it is 9.35% [1]. Most developed countries report relatively higher HUA prevalence rates, and coastal regions exhibit higher rates compared to inland areas [2,3]. In China, the prevalence of HUA is increasing annually and showing a trend toward younger populations, making it the second-largest metabolic disease after diabetes. A prospective study based on the China Kadoorie Biobank (CKB), with a median follow-up of 12.1 years involving over 510,000 Chinese adults, showed an HUA prevalence of 15.4%, with higher rates observed among males, urban residents, and the elderly [4].

Despite current research revealing the potential of various interventions in HUA management, numerous challenges remain in precise intervention and long-term management. The cornerstone of conventional HUA management involves restricting purine-rich foods (e.g., red meat), high-fructose beverages, and alcohol. Key lifestyle recommendations further emphasize weight reduction, adoption of balanced dietary patterns such as the DASH diet, adequate hydration, and intake of low-fat dairy products. Although these measures are clinically effective, long-term adherence remains challenging, and their efficacy is often insufficient in moderate-to-severe HUA, typically necessitating adjunct pharmacological therapy. Regarding pathogenesis, the interaction mechanism between genetic susceptibility and environmental factors has not been fully elucidated. In clinical intervention, there is a lack of high-level evidence supporting the synergistic effects of different uric acid-lowering strategies and personalized application schemes. Regarding efficacy evaluation, the predictive value of existing biomarkers for long-term prognosis awaits verification. Furthermore, while Traditional Chinese Medicine (TCM) interventions show advantages in multi-target regulation, the mechanisms of active ingredients and the standardization of preparations remain research difficulties. Additionally, an integrated HUA management paradigm based on “comprehensive, multi-dimensional, and personalized” approaches has not yet been maturely established.

This article aims to critically review the latest research progress on HUA prevention and treatment through organizing existing evidence from pathogenesis to clinical management. We systematically searched PubMed, Web of Science, and China National Knowledge Infrastructure (CNKI) databases up to January 2026 with the search terms including “hyperuricemia,” “uric acid,” “intervention,” “exercise,” “diet,” “gut microbiota,” and “molecular mechanisms.” We primarily included high-impact studies published within the last ten years. The exclusion criteria were (i) studies with an off-topic or non-mechanistic focus and (ii) gray literature (conference proceedings) or unpublished data. Reference lists of key articles were carefully checked to ensure comprehensive coverage of multiple interventions of HUA and molecular pathways. We hope to provide new perspectives for deepening disease cognition and optimizing prevention strategies, and offer theoretical support for promoting a new model of multifaceted HUA management based on multidisciplinary collaboration.

## 2. Overview of HUA

### 2.1. Diagnostic Criteria for HUA

HUA is a metabolic disease characterized by serum uric acid (SUA) levels exceeding the normal range [2]. Typically, HUA patients present as either symptomatic or asymptomatic [5]. Regarding diagnostic criteria, differences exist across studies and regions, but the core basis is abnormally elevated SUA levels. Currently, countries such as the USA, Japan, and Russia define HUA as SUA > 420 μmol/L for men and >360 μmol/L for women, while European standards (e.g., UK, EU) define it as SUA ≥ 404.0 μmol/L for men and ≥339.0 μmol/L for women [2]. In China, the diagnostic criteria define HUA as a fasting SUA level > 420 µmol/L for men and postmenopausal women, and >360 µmol/L for premenopausal women, measured on two different days under a normal purine diet [6]. In children and adolescents, SUA levels change with age, and while a universally accepted definition for pediatric HUA has not yet been formed, current clinical practice recommends referring to adult standards [7,8].

### 2.2. Prevalence of HUA and Its Complications

Globally, the prevalence of HUA shows a continuous upward trend with significant regional, gender, and age differences. Generally, prevalence in developed countries is higher than in developing countries. Specific data show that the prevalence of HUA in American adult males and females is 20.2% and 20.0%, respectively [9]. In Japan, the prevalence is 26.8% for men and 0.9% for women [10]. The overall prevalence rates in Australia, South Korea, the UK, and Spain are 16.6% [11], 11.4% (17.0% male, 5.9% female) [12], 27.72% (male) and 10.69% (female) [13] and 16.3% [14], respectively. Among developing countries, Bangladesh has a prevalence of 9.3% (8.4% male, 10.2% female) [15], and Thailand has 10.6% (18.4% male, 7.8% female) [16]. China faces a severe HUA situation: between 2014 and 2017, the adult prevalence rose from 13.3% to 17.7%, with male prevalence (23.5%) significantly higher than female (11.7%) [17,18]. The prevalence among adolescents is also rising rapidly, with detection rates in Chinese adolescents ranging between 26.6% and 42.3% [19]. Regionally, the prevalence in Southern China (9.1%) is higher than in Northern China (3.2%), urban areas (8.0%) are higher than rural areas (5.0%), and coastal regions (44.7%) are significantly higher than inland regions [20]. Furthermore, specific populations such as patients with type 2 diabetes mellitus (T2DM), middle to elderly gout patients, obese children and adolescents face a particularly prominent risk of HUA [21,22,23].

HUA is not an isolated metabolic abnormality. It is closely related to the development of various diseases, causing severe harm to the urinary, locomotor, endocrine, and cardiovascular systems. When SUA levels exceed saturation in HUA patients, Monosodium Urate (MSU) crystals easily precipitate. Deposition of MSU crystals in the kidneys can trigger urate nephropathy, manifested as renal tubular dilation, glomerular swelling and sclerosis, and may even progress to chronic renal failure. Additionally, HUA can further exacerbate kidney damage and increase the risk of proteinuria and kidney stones by reducing renal nitric oxide production and activating the “Renin–Angiotensin–Aldosterone” system [2,24]. If MSU deposits in joints and surrounding soft tissues, it triggers acute gouty arthritis. The long-term recurrent attacks can lead to joint deformity, bone destruction, and tophi formation [25]. HUA is also clinically associated with various musculoskeletal diseases, including sarcopenia, osteoarthritis, intervertebral disc degeneration, and osteoporosis [26]. Moreover, HUA aggravates glucose and lipid metabolism disorders, increasing the risk of T2DM [27] and cardiovascular diseases [28]. Studies have confirmed that HUA is an independent risk factor for human with T2DM; for every 1 mg/dL increase in uric acid concentration, the risk of T2DM increases by 6% [29]. It also accelerates the progression of diabetic complications such as diabetic nephropathy, diabetic retinopathy, and peripheral neuropathy [30,31,32].

The core mechanisms by which HUA induces glucose and lipid metabolism disorders are mainly reflected in two aspects: First, it affects the expression of fatty acid synthases and catabolic enzymes. Elevated uric acid inhibits hepatic PI3K/Akt signaling by inducing inhibitory phosphorylation of IRS1 (Ser312), thereby upregulating key lipogenic transcription factors (SREBP-1c, PPARγ) and enzymes (e.g., FAS, ACC), while downregulating fatty acid oxidation regulators (PPARα) and key enzymes (e.g., CPT1A). These factors ultimately lead to increased triglyceride synthesis and decreased decomposition, triggering hepatic steatosis and hypertriglyceridemia [33]. Second, it affects the insulin pathway. Both in vivo and in vitro experiments confirm that elevated uric acid significantly upregulates inhibitory phosphorylation of IRS1 (Ser312/307) in human hepatocytes (LO2) and mouse liver tissue, while downregulating phosphorylation at the key Akt activation site (Ser473), thereby impairing the hepatic insulin signaling pathway [33] and accelerating the onset and progression of diabetes.

In summary, the prevalence of HUA is rising annually and is closely related to the onset and development of multi-system injuries, including gout, chronic kidney disease (CKD), diabetes, and their complications. Therefore, early precise prevention and co-morbidity management of HUA are crucial.

## 3. Pathogenesis and Risk Factors of HUA

### 3.1. Pathogenesis

The core mechanism of HUA lies in excessive uric acid production or impaired excretion. Human uric acid is mainly derived from endogenous purine synthesis and exogenous purine intake. Abnormal activity and expression of key purine metabolic enzymes, such as Xanthine Oxidase (XO), are the central mechanisms leading to abnormal endogenous uric acid levels [2,34], while high-purine diets, such as those high in sugar and fat, are important triggers for exogenous production [35,36].

Uric acid is primarily excreted through the kidneys (approx. 70%) and the intestines (approx. 30%); renal excretion dysfunction is the main cause of HUA development [2]. Renal excretion of uric acid involves multiple steps, including glomerular filtration, renal tubular reabsorption, secretion, and post-secretory reabsorption. Functional abnormalities of transporters, including urate transporters URAT1 (SLC22A12), GLUT9/SLC2A9, and ABCG2, can directly induce HUA. For example, increased activity of URAT1, the main reabsorption transporter located on the apical membrane of renal tubules, leads to increased uric acid reabsorption. Meanwhile, ABCG2 mediates uric acid secretion, and its loss of function (e.g., rs2231142 variant) increases HUA risk. Additionally, functional abnormalities in GLUT9, OAT1 (SLC22A6), and OAT3 (SLC22A8) also affect uric acid transport [37,38]. Moreover, aging and various pathological conditions can induce HUA by hindering these processes. Elderly individuals experience reduced uric acid excretion capacity due to nephron loss [2]. Pathological conditions, such as chronic kidney disease, hypertension, and diabetes, can lead to elevated SUA by reducing the estimated glomerular filtration rate (eGFR) and enhancing renal tubular reabsorption [30]. Organic acids such as lactate and ketone bodies can competitively inhibit uric acid excretion and induce transient HUA [39]. Additionally, insulin resistance can increase uric acid reabsorption through mechanisms such as promoting URAT1 expression [2].

Additionally, abnormalities in non-renal excretion pathways including gut microbiota and Nod-like receptor family, pyrin domain containing 3 (NLRP3) inflammasome inflammation are attributed to the development of HUA. Gut microbiota participates in uric acid decomposition and excretion by producing uricase and purine oxidase. Under normal conditions, certain intestinal bacteria (e.g., *Lactobacillus*, *Pseudomonas*) can secrete uricase to decompose uric acid into allantoin, promoting excretion [2,40]. Gut microbiota dysbiosis is a major cause of intestinal uric acid excretion barriers. Dysbiosis reduces uric acid decomposition by downregulating uricase activity. Concurrently, it induces intestinal barrier impairment, accelerating endotoxin (e.g., lipopolysaccharide) entry into the blood, activating the Toll-like receptor signaling pathway, promoting the release of inflammatory factors (e.g., IL-1β, IL-6), and subsequently activating XO activity and uric acid production [2,40]. The NLRP3 inflammasome is critical to gout arthritis (GA) onset and progression. Monosodium urate crystals from HUA activate it via NF-κB-mediated transcriptional upregulation and complex assembly, stimulating the release of pro-inflammatory factors like IL-1β to drive GA’s inflammatory cascade, and it thus acts as a potential diagnostic biomarker and promising therapeutic target for GA.

### 3.2. Risk Factors

The occurrence and development of HUA are influenced by multiple factors, mainly including genetics, diet, exercise, obesity, and others. Genetic variation is an important intrinsic factor for HUA, mainly affecting uric acid metabolism and excretion processes. Clinical studies report that about 38.7% of adolescent gout patients have a family history of gout [41]. Genome-wide association studies (GWASs) report that multiple genetic association genes, such as the *ABCG2* gene, *SLC2A9* gene, and purine metabolism-related genes (e.g., *XO*, *PRPP synthetase*, and *HPRT*), are associated with HUA susceptibility [2]. Core susceptibility genes differ across races, for example, *ABCG2* variation has a more significant impact in East Asian populations, while *SLC2A9* predominates in European populations. These genes commonly participate in HUA development by enhancing uric acid reabsorption, reducing secretion or accelerating purine decomposition [2,41].

High-purine dietary intake is a significant extrinsic factor inducing HUA. Long-term high intake of high-purine foods such as animal offal and seafood increases the exogenous purine load, promoting uric acid production. Guanosine in beer and alcohol metabolites inhibit uric acid excretion [2,35,42]. Notably, the adverse effects of high fructose intake on uric acid are easily overlooked. In sugary beverages, fructose metabolism consumes ATP, activates AMP deaminase, promotes purine catabolism, and increases uric acid production, increasing the HUA risk [2,36,43]. Thus, compared to genetic variation, limiting high-purine dietary intake is a more controllable intervention. Against the backdrop of HUA increasingly affecting younger people, the impact of fructose on uric acid should be highly prioritized, especially by limiting sugar-sweetened beverage intake in children and adolescents.

HUA development is also influenced by other pathological states such as obesity. Obesity is an important independent risk factor for HUA. Clinical studies report that the risk of gout in obese individuals is 2–3 times that of normal-weight individuals [44]. The higher the degree of obesity, the higher the risk of HUA [43,45]. A recent retrospective cohort analysis in the US showed that the probability of HUA in obese populations is 2.32 times that of normal-weight populations [46]. Visceral Fat Area (VFA) is an independent risk factor for HUA, the visceral fat index and lipid accumulation product show significantly better predictive value for HUA than other obesity indicators [47]. Weight loss can reduce VFA, thereby reducing uric acid production [47]. A 5–10% weight reduction can significantly reduce the risk of HUA and gout [44]. Additionally, diseases like T2DM, CKD, and hypertension can increase HUA risk by affecting uric acid metabolism [2,31,48].

Unreasonable physical activity is closely related to HUA development. Long-term lack of physical activity induces lipid metabolism disorders and insulin resistance, thereby increasing HUA risk [39,49]. Conversely, high-intensity acute exercise or exhaustive exercise is also associated with HUA. Previous studies reported that high-intensity acute exercise leads to a transient increase in serum uric acid concentration, for instance, hypoxanthine and SUA concentrations increased by 364% and 36%, respectively, after a 100 m run, and by 1598% and 66% after an 800 m run [39]. High-load exercise promotes purine catabolism by inducing muscle damage and releasing large amounts of ATP and inflammatory factors. On the other hand, it raises blood lactate levels, which competitively inhibit uric acid excretion in renal tubules, leading to transient SUA elevation and inducing gout attacks. Especially for patients with a history of gout, this may induce joint inflammation and increase the risk of joint damage [34,39].

Furthermore, factors such as aging and the side effects from drug consumption are important risk factors. HUA prevalence increases with age, as elderly individuals have reduced uric acid excretion capacity due to declining renal function and hormonal changes [2]. Male HUA prevalence is generally higher than female; however, after menopause, female prevalence rises rapidly to approach male levels due to the sudden drop in estrogen [2]. Diuretics (e.g., hydrochlorothiazide), low-dose aspirin, and certain anti-tuberculosis drugs (e.g., pyrazinamide) can cause elevated SUA by inhibiting renal tubular uric acid excretion [2]. Additionally, tumor lysis syndrome caused by cancer chemoradiotherapy can trigger acute HUA [2].

In summary, HUA pathogenesis is influenced by multiple factors. Compared to intrinsic factors like genetics and aging, extrinsic factors such as diet, physical activity levels, and medication are important controllable factors. Choosing a reasonable, healthy, and nutritional diet while meeting daily energy needs, selecting appropriate exercise interventions while ensuring biological effects, and reducing the interference of complications in the presence of comorbidities are important directions in current health management for HUA patients.

## 4. Comprehensive Intervention Pathways for HUA

The occurrence and development of HUA and its related diseases have become significant factors endangering human life and health. Data from clinical and basic research indicate that pathways such as dietary control, lifestyle optimization, exercise intervention, and pharmacotherapy can effectively prevent and alleviate the onset and development of HUA (Table 1).

### 4.1. Dietary Intervention

Dietary intervention is the foundation of HUA management; reducing HUA-triggering foods and increasing protective foods are core aspects. Regarding triggering foods, strict restriction of high-purine foods such as animal offal, seafood, rich meat broths, and red meat can reduce SUA levels by 10–15% [2]. Avoiding sugar-sweetened beverages, alcohol (particularly beer) and high-fructose fruits can reduce the risk of obesity complicated with HUA [36,50]. However, long-term single purine restriction shows poor sustainability in gout management and may even produce harmful cardiometabolic consequences [51]. Overall pattern optimization, such as the Mediterranean diet and Dietary Approaches to Stop Hypertension (DASH), are two proven effective modes [35,42]. The Mediterranean diet alleviates HUA mainly because it is rich in vitamins and antioxidants, mitigating oxidative stress caused by purine metabolism disorders and improving gut microbiota [52]. The DASH diet emphasizes high potassium, calcium, magnesium, and fiber, with low saturated fat, added sugar, and sodium, lowering gout risk by limiting SUA production and promoting SUA excretion through metal ion regulation of cellular balance [53]. Additionally, plant-based high-purine foods (e.g., legumes, spinach, mushrooms) do not require strict restriction due to low purine bioavailability and richness in dietary fiber. Conversely, moderate intake is actually beneficial for metabolism [2]. Therefore, it should be noted that HUA dietary management needs to evolve from “single restriction of low purine” to “overall pattern optimization”.

**Table 1:** Multiple intervention strategies for HUA management.

Intervention Modality	Intervention Protocol	Target Population	Clinical Outcomes	Changes in HUA-Associated Diseases	Ref.
Mediterranean Diet	Calories: Female ≤ 1500 kcal/d, Male ≤ 1800 kcal/d Fat-to-energy ratio < 35% Duration: 2 years	52.7 ± 6.3 years	BP ↓ 6 months: Weight ↓ 5.1 kg, SUA ↓ 119 μmol/L 24 months: Weight ↓ 4.5 kg, SUA ↓ 83 μmol/L	Insulin ↓ Improved lipid profile and glomerular filtration	[52]
DASH Diet	Energy intake levels: 1600–3100 kcal; Pattern: High K/Mg/Ca, low fat/sugar. Duration: 8 weeks.	45 ± 1 years	SUA ↓ 0.25 mg/dL BP ↓ LDL-C ↓	Improvement in hypertension and hyperlipidemia.	[53]
Baduanjin	Low-to-moderate intensity 45 min/session, 4 sessions/week. Duration: 12 weeks	Gout patients (55–75 years)	SUA ↓ 45.86 μmol/L Blood glucose ↓ 0.71 mmol/L; BP ↓; BMI ↓.	Improvement in gout symptoms.	[54]
Baduanjin	40 min/session 1 session/day. Duration: 6 months	50.2 ± 7.8 years	SUA ↓ 55.67 μmol/L BMI ↓ 1.67 kg/m^2^ Blood glucose ↓ 0.78 mmol/L Lipids ↓.	Reduced risk of hyperglycemia, diabetes, and cardiovascular disease.	[55]
24-Form Tai Chi	60 min/session 2 sessions/week. Duration: 3 months	Patients with metabolic syndrome (58.5 years)	SUA ↓ 66.27 μmol/L BMI ↓ 1.3 kg/m^2^ Blood glucose ↓ 0.61 mmol/L Lipids ↓ 0.94 mmol/L DBP ↓ 4.24 mmHg	Reduced cardiovascular risk; Improvement in metabolic syndrome components.	[56]
Aerobic Exercise	Brisk walking or jogging at 55–70% VO_2_max; 40 min/session, 3 sessions/week. Duration: 24 weeks.	41.9 ± 15.7 years	SUA ↓ 150.81 μmol/L Weight ↓ 5.7 kg BMI ↓ 3.8 kg/m^2^.	Improved lipid metabolism.	[57]
Resistance Training	10 reps/20RM; 40–50 min/session, 3 sessions/week. Duration: 24 weeks.	41.6 ± 16.4 years	SUA ↓ 143.45 μmol/L Weight ↓ 4.48 kg; BMI ↓ 2.65 kg/m^2^.	Improved lipid metabolism	[57]
Aerobic Exercise	55–75% HRmax 30–40 min/session 3–4 sessions/week Duration: 3 months.	Mean age: 48 years	SUA ↓ 193 μmol/L Weight ↓ 9.6 kg BMI ↓ 2.0 kg/m^2^.	Improved lipid metabolism; Gout flare frequency ↓	[58]
Aerobic Exercise	Moderate intensity PA: 3.0–6.0 METs.	Obese population (47.2 ± 4.4 years)	For every 1 MET-h/day increase, SUA ↓ 13.2 μmol/L.	—	[59]
Resistance Training	50–70% 1RM 3 sets × 15 reps/exercise 1 min rest interval; 60 min/session. Duration: 8 weeks.	26.3 ± 3.6 years	Post-training SUA ↑ Magnitude of SUA increase (Δ%) ↓	—	[60]
High-Intensity Resistance Training	5 sets × 10 reps at 70% 1RM 90 s rest interval 60 min/session	22.7 ± 0.6 years	SUA ↑ within 4 h (Acute response).	—	[61]
Resistance Training	3 sets × 12RM × 7 exercises 2 min rest interval; 3 sessions/week. Duration: 12 weeks.	T2DM patients (58.94 ± 10.65 years)	SUA ↑	—	[62]
Speed-Strength + Aerobic	Speed-strength: 15–20 RM, 1–3 min rest Aerobic: 50–65% HRmax, 40 min/session; 4 sessions/week. Duration: 12 weeks.	Obese patients (35.00 ± 8.14 years)	SUA ↓ 153.33 μmol/L Weight ↓ 6.53 kg BMI ↓ 2.13 kg/m^2^.	Improved lipid metabolism; Reduced obesity severity; Cardiovascular risk ↓	[63]
Speed-Strength + Aerobic Dance	Intensity: 40–60% HRmax; Resistance: 3 sets × 10 reps. 30–40 min/session, 3 sessions/week. Duration: 12 weeks.	Males (38.45 ± 8.56 years)	SUA ↓ 141.45 μmol/L.	Improved lipid metabolism, hypertension, and gout symptoms.	[64]
Aerobic + Resistance	Aerobic: 30 min; Resistance: 15–20 min at 60–70% 1RM. 60 min/session, 3–5 sessions/week. Duration: 12 weeks.	50.1 ± 11.2 years	SUA ↓ 84.92 μmol/L.	Improved lipid metabolism.	[65]
Aerobic + Resistance	Aerobic (30 min): 60–70% VO_2_max; Resistance (20 min). 50 min/session, 3 sessions/week. Duration: 12 weeks.	20.60 ± 1.57 years	SUA ↓ 122.00 μmol/L; BMI ↓ 1.85 kg/m^2^ VO_2_max ↑ 220.45 mL/min.	Improved lipid metabolism.	[66]
Moderate Aerobic + Strength	Details not specified.	69.5 ± 1.9 years	SUA ↓ 266.77 μmol/L Resting HR ↓ 9.25 bpm	—	[67]
Aerobic + Strength	Aerobic (20–30 min): 40–60% HRmax; Strength (15–20 min). 3 sessions/week. Duration: 12 weeks.	66.47 ± 5.36 years	SUA ↓ 147.84 μmol/L VO_2_max ↑ 1500 mL/min.	Improved renal function.	[68]
Topiroxostat vs. Allopurinol	Topiroxostat: 40 mg/d, max 160 mg/d; Allopurinol: 100 mg/d, max 200 mg/d. (Dose increased every 4 weeks).	CHF patients with HUA (70–71 years)	Reduction in Topiroxostat group was significantly greater than Allopurinol group (−2.7 ± 1.5 > −2.2 ± 1.2 mg/dL).	—	[69]
Allopurinol vs. Febuxostat	Allopurinol: Start 100 mg/d, max 800 mg/d; Febuxostat: Start 40 mg/d, max 80 mg/d.	Mean age: 62.1 years	SUA decreased in both groups.	—	[70]
Empagliflozin vs. Benzbromarone	Empagliflozin: 25 mg q.d.; Benzbromarone: 100 mg q.d.	T2DM patients	After 1 week: Empagliflozin ↓ 1.0 mg/dL; Benzbromarone ↓ 3.5 mg/dL.	SGLT2 inhibitors improved urinary glucose excretion.	[71]
Probenecid + Colchicine	Probenecid 500 mg b.i.d. + Colchicine 0.5 mg b.i.d. vs. Colchicine 0.5 mg b.i.d. (with placebo). Duration: 2 weeks.	Acute gouty arthritis patients	Acute phase: Probenecid + Colchicine combination reduced serum uric acid > Colchicine alone.	—	[72]

Notes: In this table, ↑ indicates an increase and ↓ indicates a decrease.

### 4.2. Gut Microbiota Intervention

Gut microbiota plays a key role in maintaining uric acid balance. Anaerobic bacteria possessing purine degradation gene clusters are widely present in the intestine and can metabolize uric acid under anaerobic conditions, providing an important intestinal uric acid clearance pathway for humans lacking uricase. Specific flora (e.g., *Clostridium* species) can directly degrade approximately thirty percent of intestinal uric acid. Probiotics exhibit uric acid-lowering and anti-inflammatory potential through mechanisms such as inhibiting XO, increasing short-chain fatty acids (SCFAs), enhancing the intestinal barrier, and regulating uric acid transporters [73,74].

Gut microbiota dysbiosis can destroy the intestinal barrier and promote inflammation and uric acid production through endotoxemia [42], closely relating to HUA. The gut microbiota structure of HUA patients is generally disordered, characterized by increased abundance of Bacteroidetes and Verrucomicrobia, decreased abundance of Firmicutes and Tenericutes, accompanied by a reduction in beneficial bacteria like *Lactobacillus* and an increase in certain pathogenic bacteria. Preclinical studies in mice have shown that clearing anaerobic bacteria with purine degradation gene clusters leads to elevated blood uric acid, while supplementing purine-degrading bacteria can reverse this change. Clinical studies also suggest that the abundance of purine degradation-related genes decreases in the gut of gout patients, and the use of antibiotics targeting anaerobes leads to increased fecal uric acid concentration, further confirming the key regulatory role of gut microbiota in uric acid homeostasis [75,76,77]. Gut microbiota regulation is expected to be a novel frontier in HUA management. The use of uricosuric probiotics or selective gut URAT1 inhibitors (e.g., topiroxostat) are emerging areas of research. Diagnostic models based on microbiota characteristics have shown potential superiority over blood uric acid testing, and fecal microbiota transplantation (FMT) effectively lowers blood uric acid in animal models, suggesting potential therapeutic value [42]. In the future, intervention strategies utilizing artificial intelligence screening and strain engineering technologies targeting gut microbiota are expected to be the important direction for precise HUA management.

In summary, gut microbiota show promise in HUA intervention through uric acid degradation, inflammation regulation, and intestinal barrier maintenance, serving as a promising supplement to traditional therapies. However, current evidence is predominantly derived from correlational or animal studies, leaving key strains, causal mechanisms, and long-term efficacy unclear. It is prudent to interpret these results with caution before translating them into clinical practice. Future research should leverage multi-omics and artificial intelligence to identify core functional microbiota and advance precise, individualized intervention strategies targeting gut microbiota.

### 4.3. Lifestyle Intervention

Lifestyle interventions mainly include popularizing knowledge about HUA etiology, hazards, and management to patients through health education lectures, distributing health management manuals, and one-on-one guidance, thereby improving patients’ Knowledge, Attitude, and Practice (KAP) regarding dietary control. Previous evidence shows that health education based on behavioral medicine theory can help patients establish long-term healthy living habits and improve intervention compliance [40]. Additionally, attention must be paid to tobacco intake and healthy sleep schedules for HUA patients. Smoking aggravates oxidative stress and inflammatory responses, increasing the risk of HUA and complications [2]; staying up late and irregular sleep schedules affect endocrine and metabolic functions, exacerbating insulin resistance and uric acid metabolism disorders [2,34]. Health education combined with exercise intervention can significantly improve dietary KAP scores in gout patients [54].

### 4.4. Exercise Intervention

As an important non-pharmacological management tool for HUA, exercise intervention has been proven in multiple studies to have significant effects in alleviating HUA and complication symptoms, with different exercise modalities showing varied effects [5,34,78].

#### 4.4.1. Traditional Health Exercises

Traditional health exercises such as Baduanjin (Eight-Section Brocade), as low-impact exercises, regulate Qi and blood and improve metabolism, making them particularly suitable for middle-aged, elderly, and gout patients. A 12-week Baduanjin intervention significantly reduced SUA levels in HUA patients, alleviated gout symptoms, and improved metabolic indicators such as blood pressure, blood glucose, and waist-to-hip ratio [54]. Hou Huiqing et al. compared Baduanjin with brisk walking in middle-aged HUA patients for 6 months, finding that Baduanjin was superior in lowering uric acid [55]. TCM exercise prescriptions for patients with metabolic syndrome complicated by HUA showed significant improvements in metabolic indicators, quality of life, and psychological state after 3 months, suggesting efficacy and favorable safety profiles [56]. Furthermore, personalized interventions for HUA patients with different TCM constitutions (e.g., Damp-Heat type, Qi-Deficiency type) have shown initial success. Studies report that in overweight/obese patients with asymptomatic HUA, BMI and WHR are significantly correlated with SUA levels across different constitution types [79]. Patients with Phlegm-Dampness constitution have higher visceral fat areas and inflammatory factor levels, with significantly increased HUA risk [80]. TCM constitution-based exercise prescriptions (e.g., Baduanjin and Tai Chi for Phlegm-Dampness; brisk walking and swimming for Damp-Heat) can significantly lower SUA levels alongside BMI and WHR reductions [56]. However, personalized Baduanjin movement adjustments, dosage optimization, and the efficacy differences between Baduanjin and other traditional exercises (like Tai Chi) in HUA intervention [54,56] remain to be further explored.

#### 4.4.2. Aerobic Exercise

Conventional low-to-moderate intensity activities such as walking, stair climbing, jogging, and swimming play a positive role by improving body composition, reducing visceral fat, and promoting uric acid excretion. Studies show that walking intervention effectively lowers blood uric acid levels and improves BMI [54]; daily stair climbing is also significantly associated with reduced HUA risk [81]. For obese patients with HUA, aerobic exercise significantly improves body composition, alleviates symptoms, and enhances overall metabolic health [45,57]. Combining low-to-moderate intensity aerobic exercise with dietary control reduces gout patients’ blood uric acid levels and attack frequency, and improves weight and lipid indicators more significantly than dietary intervention alone [58]. Regarding intensity, moderate-intensity aerobic exercise has the most definitive health promotion effects on HUA; research indicates its uric acid-lowering effect is superior to low-intensity exercise and sedentary controls [59]. However, limitations exist: exercise parameters (duration, frequency) and comparative effects of different modes for obese HUA populations are unclear [39,63], safety and appropriate intensity for HUA patients with complications like CKD require further study [39,78].

#### 4.4.3. Resistance Exercise

The impact of resistance exercise on uric acid levels remains controversial. Some studies report that resistance training, including rapid strength training, reduces uric acid production by enhancing muscle strength, endurance and improving oxidative stress and inflammation [49,64]. Others suggest high-intensity resistance exercise can raise uric acid levels in healthy individuals [60,61]. A 12-week heavy-load resistance training program significantly increased uric acid levels in T2DM patients, primarily because elevated lactate levels under anaerobic glycolysis inhibited uric acid excretion [62]. The divergent results may stem from differences in intensity and rest intervals. Moderate-intensity resistance training with sufficient rest intervals effectively lowered blood uric acid in gout patients [57]. Therefore, compared to aerobic exercise, resistance exercise carries a risk of raising uric acid; thus, lower intensity and sufficient intervals should be selected based on individual conditions. Additionally, low-load resistance training schemes for elderly sarcopenia patients with HUA require optimization through more research [47].

#### 4.4.4. Combined Exercise

Combined exercise, typically the integration of aerobic and resistance training, has been widely confirmed to synergistically improve metabolic health through multiple pathways, with effects generally superior to single exercise modes [45,82]. This form of intervention effectively alleviates oxidative stress and inflammation and regulates uric acid metabolism, thereby lowering blood uric acid [49,64,65]. Studies show positive impacts across HUA patients with different health statuses. In HUA populations with obesity or T2DM, aerobic plus resistance intervention showed particularly obvious effects in improving gout symptoms and metabolic indicators [63]. In college students, 12 weeks of combined training was superior to Baduanjin or health education alone in lowering SUA and improving body composition and cardiorespiratory fitness [66]. For elderly HUA patients, moderate-intensity aerobic plus strength training not only significantly reduced SUA and improved resting heart rate, vital capacity, and quality of life [67], but may also enhance renal uric acid excretion function [68]. Regarding intensity, most studies use low-to-moderate intensity to balance efficacy and safety, avoiding uric acid fluctuations caused by lactate accumulation [82]. Some studies also suggest High-Intensity Interval Training (HIIT) combined with dietary adjustment has potential to improve SUA, lipids, and islet function [83]. In summary, existing evidence supports the overall advantage of combined exercise [45,82]. However, relative efficacy of different combinations, optimal parameters (intensity, frequency, duration) for specific populations (e.g., those with T2DM), and exact biological mechanisms await systematic elucidation via large-sample, multi-center randomized controlled trials [39,54,78].

### 4.5. Pharmacological Intervention

For HUA patients with complications like gout, pharmacotherapy is usually a core intervention. Current drugs fall into three categories: uric acid synthesis inhibitors (e.g., Allopurinol, Febuxostat, Topiroxostat), uricosuric agents (e.g., Benzbromarone, Probenecid, SGLT2 inhibitors), and uric acid metabolic enzymes (e.g., Rasburicase, Pegloticase). Low-dose colchicine or NSAIDs are used during initial First-Line urate-lowering therapy to prevent gout flares driven by the NLRP3 inflammasome activation upon mobilization of urate crystals. Studies show that in gout patients with CKD, Allopurinol and Febuxostat have similar efficacy and tolerance under a treat-to-target strategy, with lower gout flare rates in the Allopurinol group [84]. In chronic heart failure patients with HUA, Topiroxostat and Allopurinol have similar effects [69]. Although Febuxostat has a stronger uric acid-lowering effect, its risk of inducing acute gout flares during early treatment is comparable to Allopurinol when combined with prophylactic anti-inflammatory therapy [70]. This suggests clinical practice should focus on standardized dose titration and flare prevention rather than simply selecting or avoiding a specific drug class. Among uricosuric drugs, SGLT2 inhibitors indirectly enhance uric acid excretion by promoting urinary glucose and sodium excretion [71]. In male gout patients with renal uric acid underexcretion, low-dose Benzbromarone showed superior uric acid-lowering effects to low-dose Febuxostat with similar favorable safety profiles [85]. Additionally, Probenecid combined with Colchicine during acute gouty arthritis was reported to lower blood uric acid levels faster without aggravating pain, facilitating early uric acid control [72].

In summary, despite an expanding pharmacopeia for HUA, key clinical hurdles remain: imprecise drug personalization, unresolved long-term safety concerns, and a lack of optimized strategies for comorbid patients. The future roadmap entails a shift towards precision medicine, leveraging pharmacogenomics and patient-specific traits to guide therapy. Achieving this requires rigorous long-term research into novel drugs and combinations, ultimately transitioning HUA management from mere uric acid control to personalized, comprehensive comorbidity management.

## 5. Mechanisms of Various Interventions in Improving HUA

**Figure 1:**
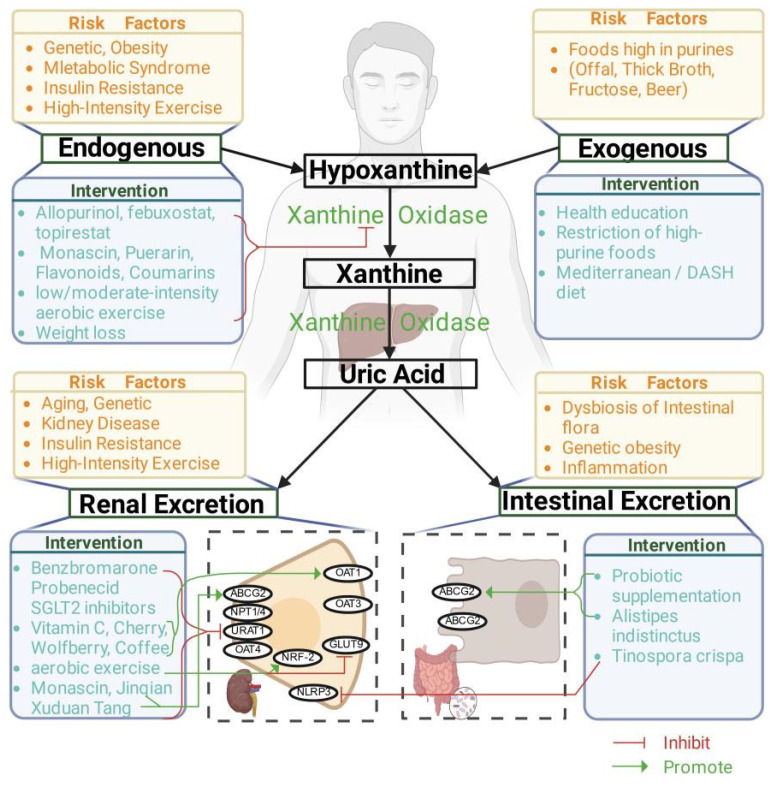
Mechanisms behind the multiple intervention strategies for HUA management. Notes: This figure is created using icons and templates from Biorender (https://www.biorender.com/. Accessed on 23 December 2025) with a valid Confirmation of Publication and Licensing Rights (Agreement Number: OV297VKL2U). Abbreviations: OAT1: SLC22A6; GLUT9:SLC2A; ABCG2: BCRP; NPT1: SLC17A1; URAT1: SLC22A12.

Multiple intervention strategies show different efficacy on HUA management through various mechanisms involved in the uric acid production and excretion process (Figure 1).

### 5.1. Molecular Mechanisms of Dietary Intervention

Dietary intervention is a basic measure for HUA management; its mechanisms mainly include influences on purine oxidase and transporter activity, gut microbiota, and glycolipid metabolism.

Regarding purine oxidase and transporter activity, limiting high-purine and fructose intake reduces endogenous uric acid synthesis induced by fructose metabolism by directly lowering the exogenous purine load [2,43]. Simultaneously, multiple studies, primarily based on animal models, have shown that certain protective foods interfere with uric acid metabolism through multi-target synergistic effects: for example, foods rich in polyphenols and alkaloids can inhibit XO activity to reduce uric acid production; vegetables and fruits rich in Vitamin C and wolfberry (Goji) can promote renal uric acid excretion by competitively inhibiting URAT1 or activating OAT1 transporters. Probiotic intervention can inhibit XO activity and regulate the activity of uric acid transporters ABCG2, GLUT9, and URAT1 [86], ultimately improving uric acid metabolism.

From the perspective of gut microbiota, gut microbiota regulate uric acid homeostasis through three main pathways [42,87]: First, preclinical studies indicate that intestinal anaerobes (especially Firmicutes) utilize conserved gene clusters (*xdhAC*) to efficiently degrade uric acid into lactate and Short-Chain Fatty Acids (SCFAs) under anaerobic conditions, thereby enhancing intestinal uric acid degradation capacity and serving as a potential compensatory mechanism for human uricase deficiency [76,77]. These SCFAs contribute to intestinal barrier integrity and anti-inflammatory effects [87]. Separately, gut microbiota-derived metabolites, such as hippuric acid, have been shown to specifically upregulate intestinal ABCG2 expression, enhancing urate excretion [87]. Second, unlike direct degradation by anaerobes, studies in rats have demonstrated that *lactobacillus* mainly reduces intestinal purine absorption at the source by competitively taking up and consuming dietary purine nucleosides [88]. Third, microbial metabolites interact closely with host transporters, as preclinical evidence in mice indicates that hippuric acid derived from specific flora (e.g., *Alistipes indistinctus*) can specifically induce the expression and localization of ABCG2 protein at the apical membrane of intestinal epithelial cells, significantly promoting intestinal uric acid excretion [89].

Regarding regulation of glucose–lipid metabolism and inflammatory states, evidence from clinical studies indicates that HUA patients’ gut microbiota exhibit a pro-inflammatory/anti-inflammatory imbalance, manifested as depletion of uric acid-degrading bacteria in Firmicutes and elevated levels of potential pathogens like Bacteroides and Enterobacteriaceae. This imbalance exacerbates metabolic disorders and inflammation. Dietary intervention improves glucose–lipid metabolism and inflammation levels, accompanied by HUA improvement. For instance, fermented foods containing specific probiotics can supplement beneficial bacteria and inhibit opportunistic pathogens. High-fiber/plant protein foods provide substrates for beneficial bacteria to maintain balance. SCFAs produced by beneficial bacteria enhance intestinal barrier integrity by upregulating tight junction protein expression, thereby reducing endotoxin translocation into the bloodstream. This suppression of endotoxemia inhibits the TLR4–NF-κB inflammatory pathway, alleviates systemic inflammation, and indirectly improves uric acid metabolism [40]. Additionally, they can mitigate inflammation and HUA symptoms by promoting neutrophil apoptosis and inhibiting histone deacetylases to down-regulate NLRP3 activity, reducing the release of pro-inflammatory factors IL-1β and TNF-α [35,42]. Furthermore, active ingredients in TCM and dietary patterns like the Mediterranean and DASH diets show promise in promoting uric acid excretion by controlling weight and improving insulin resistance and lipid metabolism [2,35,42,86].

In summary, dietary intervention modulates HUA by targeting key pathways: regulating uric acid-metabolizing enzymes and transporters, restoring gut microbiota balance, and attenuating inflammatory responses. Specific mechanisms involve anaerobic bacterial degradation of uric acid into SCFAs, *Lactobacillus*-mediated purine absorption interception, hippuric acid-induced upregulation of ABCG2 excretion, synergistic intestinal barrier repair, and suppression of NLRP3/TLR4 inflammatory signaling. However, several challenges remain, including conflicting mechanistic hypotheses (e.g., allantoin vs. SCFA pathways), difficulties in culturing and colonizing novel purine-degrading bacteria (PDB), and a lack of large-scale clinical validation. These factors contribute to unstable efficacy and limited translational application. Current evidence relies heavily on animal and observational studies, with insufficient high-quality clinical trials and limitations such as significant individual variability and inconsistent microbiota analytical methods [86,87]. Future prospective randomized controlled trials are needed to clarify the clinical efficacy and mechanisms of diet and probiotic interventions, promoting the development of precision nutrition strategies.

### 5.2. Molecular Mechanisms of Exercise Intervention

#### 5.2.1. Regulating Uric Acid Synthesis, Transport, and Decomposition Functions

Exercise intervention can affect uric acid synthesis, transport, and decomposition through various ways including downregulation of the activity and protein expression of the rate-limiting enzyme XO in the kidneys. Previous studies noted that ATP decomposition accompanying strenuous exercise accumulates large amounts of substrates like hypoxanthine, driving XO to produce excessive Reactive Oxygen Species (ROS) and trigger oxidative stress [90]. Whereas regular exercise intervention produces adaptive changes, reducing XO activity and mitigating oxidative stress associated with uric acid production at the source [91]. Thus, exercise intensity plays a key threshold role. Zhang et al. [92] pointed out that high-intensity exercise leads to phosphocreatine depletion and massive ATP decomposition, accelerating purine metabolite generation. Further research confirmed that strenuous or competitive training prompts the body to accumulate massive hypoxanthine, the increased substrate concentration conversely activates XO activity, leading to synchronized elevation of blood uric acid and oxidative stress markers [90,93]. Notably, single bouts of anaerobic exhaustive exercise have a significant “delayed” activation effect on XO, where oxidative stress peaks 24 h post-exercise, a reaction more intense in individuals with poor anaerobic endurance [94]. Therefore, the inhibitory effect of long-term aerobic exercise on XO is likely reflected in adaptive low-to-moderate intensity loads. Regarding molecular regulation of renal excretion, Feoli et al. [95] found XO activity significantly positively correlated with systemic inflammatory status, suggesting XO is not just a metabolic enzyme but an inflammatory mediator. Based on this, exercise affects uric acid metabolism by regulating transporter activity and expression. Long-term aerobic exercise generates circulating factors that significantly upregulate the expression of the key uric acid secretion transporter ABCC4 (MRP4) in renal tubular epithelial cells by activating the antioxidant signal pathway NRF-2 and inhibiting the pro-inflammatory pathway NF-κB. Although this process also causes a compensatory increase in reabsorption proteins URAT1 and GLUT9, the ABCC4-mediated excretion enhancement dominates, effectively promoting renal uric acid excretion and lowering serum levels [96]. Exercise also promotes excretion by improving uric acid transport function [54] and increasing renal blood flow to improve glomerular filtration [34].

#### 5.2.2. Improving Body Composition and Insulin Resistance

Obesity leads to adipose tissue secreting inflammatory factors, elevating XO activity, and promoting hepatic uric acid production [97]. Obesity accompanied by insulin resistance jointly raises HUA risk. Weight loss reduces inflammatory factors secreted by visceral fat, inhibits the NF-κB pathway, lowers XO activity, and reduces uric acid production [44,47]. Exercise reduces uric acid production and excretion barriers by increasing energy expenditure, reducing fat accumulation, and improving metabolic load [54,63,64,98]. Zhang Huabing et al. found that reduced VFA decreases free fatty acid release, inhibits XO activity, and reduces uric acid production. The increased skeletal muscle mass reduces URAT activity and renal tubular uric acid reabsorption by improving insulin resistance [47]. Additionally, exercise reduces non-HDL cholesterol and lipotoxicity by improving lipoprotein abnormalities, alleviating hepatic lipid deposition and inflammation, and reducing the metabolic disorder state affecting uric acid metabolism, thereby lowering HUA risk [98]. Exercise improves insulin sensitivity, subsequently improving the regulation of renal proximal tubule uric acid transporters (e.g., URAT1/GLUT9) and promoting excretion, a key pathway for exercise-induced HUA reduction [98].

#### 5.2.3. Regulating Oxidative Stress and Inflammatory Response

Inflammation is a key pathological mechanism of HUA and exercise plays an important role in improving systemic inflammation [99]. Exercise inhibits inflammatory responses by reducing the release of inflammatory signaling factors from neutrophils and monocytes, reducing the negative impact of the pro-inflammatory environment on the renal uric acid transport system, thereby lowering blood uric acid [98]. Rapid strength training combined with aerobic exercise reduced Malondialdehyde (MDA) levels and IL-6/TNF-α levels while increasing SOD activity in obese HUA patients [64]. Furthermore, exercise-related muscle mass increase indirectly regulates uric acid metabolism by reducing ROS generation and alleviating oxidative stress [47,64].

In summary, exercise synergistically improves HUA through multiple pathways: inhibiting XO activity, regulating uric acid transporter expression, reducing fat accumulation, and improving insulin resistance and inflammatory responses. However, the direct molecular regulation mechanisms of exercise on uric acid levels are not yet fully elucidated, and research in this area remains insufficient. Most current studies have limited sample sizes and lack randomized controlled trials. Future research requires large-sample randomized controlled trials on different exercise intervention modes, intensities, and cycles across HUA populations with varying health degrees.

### 5.3. Molecular Mechanisms of Pharmacological Intervention

#### 5.3.1. Mechanisms of Uric Acid Synthesis Inhibitors

Uric acid production relies on XO catalyzing purine metabolism. Intervention reducing uric acid synthesis centers on inhibiting XO activity and regulating purine metabolic pathways. Animal experiments show that Monascus yellow pigments significantly inhibit XO activity, block the uric acid production pathway, and lower blood uric acid, the high-dose group showed effects comparable to allopurinol without hepatorenal toxicity [100]. The formulas of the puerarin-rich compound Puerariae lobatae and their active ingredient puerarin significantly reduced blood uric acid in HUA mice through regulating purine metabolism-related pathways, and improving liver/kidney function and inflammation [101]. While these findings reveal a clear mechanism of action in vivo, clinical trials are needed to confirm the dose and efficacy in human patients. Furthermore, studies have systematically elucidated the potential of natural products as XO inhibitors, flavonoids, coumarins, and alkaloids interfere with key enzymes in uric acid production through multi-target synergistic effects. This is a key current research direction, aligning with classic research on XO structure and inhibitor modes, providing biochemical support for natural product inhibitors [102].

#### 5.3.2. Mechanisms of Uricosuric Agents

Uric acid excretion mainly relies on renal filtration and urinary discharge, finely regulated by a series of transporters including URAT1 and GLUT9 (reabsorption) and OAT1/3 and ABCG2 (secretion). The expression and functional status of these transporters directly affect blood uric acid levels and are core targets for uricosuric drug development [38]. Research finds that various natural products promote excretion by regulating these key transporters. For example, *Tinospora crispa* vine extract promotes excretion by regulating purine metabolism and targets like STAT3 and PPARG [103]; cyclodextrin and its polymers improve excretion efficiency by complexing uric acid and inhibiting crystal formation [104]. Monascus yellow pigments promote excretion by downregulating URAT1/GLUT9 and upregulating ABCG2, while improving oxidative stress and inflammation [100]. Active ingredients in Jinqian Xuduan Decoction regulate targets like URAT1 and GLUT9, exerting anti-oxidative and anti-fibrotic effects while promoting excretion [105]. These findings are built on solid research into uric acid transporter function, with numerous in vivo/in vitro studies clarifying the key roles of URAT1, GLUT9, OAT1/3 in reabsorption, secretion and their significance in drug response [37,106].

In summary, the pharmacological reduction of uric acid hinges on two principal strategies: suppressing production via XO inhibition and enhancing excretion through blockade of URAT1/GLUT9 or modulation of SGLT2. Nevertheless, despite considerable mechanistic insight, the evidence base for these targets varies across intervention modalities (Table 2), a critical factor for clinical application. Persistent knowledge gaps include the precise structural basis of transporter selectivity, the system-wide metabolic adaptations to drug intervention, and the mechanistic synergy of combinatorial regimens. Advancing the field will necessitate integrated functional studies and multi-omics technologies to fully map these action pathways.

## 6. Conclusions and Perspectives

Research on comprehensive interventions for HUA has gradually expanded from traditional dietary management and pharmacotherapy to multi-dimensional approaches, including exercise therapy, lifestyle optimization, and gut microbiota regulation. Each intervention demonstrates distinct biological roles in regulating uric acid production, improving excretion, and correcting metabolic and inflammatory environments. Dietary interventions manage the health of HUA patients by regulating the activity of oxidases and transporters related to uric acid metabolism, correcting gut microbiota dysbiosis, and reducing inflammatory states. Exercise interventions synergistically improve uric acid homeostasis through multiple mechanisms, such as regulating the activity of enzymes related to purine metabolism, improving body composition, and alleviating insulin resistance and oxidative stress. Pharmacotherapy remains a core measure for patients with severe conditions, exerting direct effects by inhibiting uric acid production or promoting excretion. However, existing research still faces significant limitations: exercise interventions lack standardized protocols due to high heterogeneity in intensity, duration, and target populations; most evidence derives from observational or small-scale randomized trials, limiting causal inference in key molecular pathways; and the synergistic effects and long-term safety of combined strategies remain unclear.

Additionally, care must be exercised when interpreting the current body of evidence. Due to the potential overlap in study populations and the heterogeneity of clinical designs, establishing universal ‘no-effect’ safety thresholds remains challenging. Consequently, clinical recommendations cannot be generalized across all demographics. Given the trend of younger onset, management for children and adolescents requires special attention. Adolescent HUA represent a distinct clinical entity marked by severe hyperuricemia, strong genetic predisposition, and polyarticular onset, carrying significant health risks. Current management emphasizes stricter, risk-based urate-lowering therapy criteria and lower treatment targets. However, evidence primarily relies on observational studies and small-sample RCTs. Therefore, future efforts should focus on conducting multi-center, large-sample, and mechanism-oriented studies, and constructing individualized health management plans based on population characteristics to further enhance the effectiveness of precision interventions for HUA.

## Figures and Tables

**Table 2:** Summary of key molecular targets of different HUA lifestyle intervention modalities and evidence.

Intervention Modalities	Specific Intervention	Key Target/Pathway	Evidence Level	Remarks	Ref.
Nutrition	foods with low purine	load of exogenous purine	Strong (clinical RCT)	Asymptomatic HUA	[2]
	DASH diet	improved insulin resistance Inhibited URAT1	Strong (clinical RCT)	Adults with elevated blood pressure	[53]
	Mediterranean Diet	Mitigated Oxidative Stress (ROS) Improved gut microbiota	Strong (clinical RCT)	T2DM or Moderate Obesity Group	[52]
	Foods rich in polyphenols/alkaloids	Inhibited xanthine oxidase (XOR) activity	Low (pre-clinical study)	Animal Models	[102]
	Vitamin C, Wolfberry	Competitively inhibited URAT1; Activated OAT1	Low (pre-clinical study)	Animal Model	[86]
	Probiotics	Inhibited XOR activity; Regulated ABCG2, GLUT9, and URAT1	Low (pre-clinical study)	Correlational or animal studies	[86]
Gut Microbiota	*Anaerobic bacteria* (e.g., Firmicutes)	Degraded uric acid via *xdhAC* gene cluster	Low (pre-clinical study)	Animal Model	[76,77]
	*Alistipes indistinctus*	Induced ABCG2 expression and localization	Low (pre-clinical study)	Animal Model	[89]
	*Lactobacillus*	Reduced dietary purine nucleoside absorption	Low (pre-clinical study)	Animal Model	[88]
Exercise	aerobic exercise	Upregulated ABCC4 via NRF-2 activation; Reduced XOR activity	Low (pre-clinical study)	Animal Model	[96]
	Weight loss/Reduced VFA	Inhibited NF-κB and XOR activity; Reduced URAT1 activity	Medium (clinical cohort)	Obese population	[44,47]
	Acute/High-intensity exercise	Activated XOR (delayed) via ATP breakdown	Medium (clinical observation)	Healthy adults/Athletes	[92]
	HIIT	Improved insulin resistance; Improved lipid metabolism	Medium (clinical study)	Patients with HUA and Gout	[83]
Pharmacological	Allopurinol/Febuxostat	Inhibited XOR activity	Strong (clinical RCT)	Gout patients with CKD or Heart Failure	[69,70,84]
	Benzbromarone	Inhibited URAT1 reabsorption	Strong (clinical RCT)	Male gout patients with renal uric acid underexcretion	[38]
	SGLT2 Inhibitors	Promoted urinary glucose and uric acid excretion	Strong (clinical RCT)	Patients with T2DM	[71]
	Monascus yellow pigments	Inhibited XOR; Downregulated URAT1/GLUT9; Upregulated ABCG2	Low (pre-clinical study)	Animal Model	[100]
	*Tinospora crispa* vine extract	Regulated STAT3 and PPARG	Low (pre-clinical study)	Animal Model	[103]
	Cyclodextrin polymers	Complexed uric acid; Inhibited crystal formation	Low (pre-clinical study)	in vitro and Animal Model	[104]
	Jinqian Xuduan Decoction	Regulated URAT1 and GLUT9	Low (pre-clinical study)	Animal Model	[105]

## Data Availability

No new data were created or analyzed in this study. Data sharing is not applicable to this article.
